# Cranial deformation and genetic diversity in three adolescent male individuals from the Great Migration Period from Osijek, eastern Croatia

**DOI:** 10.1371/journal.pone.0216366

**Published:** 2019-08-21

**Authors:** Daniel Fernandes, Kendra Sirak, Olivia Cheronet, Rachel Howcroft, Mislav Čavka, Dženi Los, Josip Burmaz, Ron Pinhasi, Mario Novak

**Affiliations:** 1 Department of Evolutionary Anthropology, University of Vienna, Vienna, Austria; 2 School of Archaeology, University College Dublin, Dublin, Ireland; 3 CIAS, Department of Life Sciences, University of Coimbra, Coimbra, Portugal; 4 Department of Genetics, Harvard Medical School, Boston, MA, United States of America; 5 Department of Diagnostic and Interventional Radiology, University Hospital Center Zagreb, Zagreb, Croatia; 6 Kaducej Ltd., Split, Croatia; 7 Institute for Anthropological Research, Zagreb, Croatia; University at Buffalo - The State University of New York, UNITED STATES

## Abstract

Three individuals dating to the Great Migration Period (5^th^ century CE) were discovered in a pit at the Hermanov vinograd site in Osijek, Croatia. We were inspired to study these individuals based on their unusual burial context as well as the identification of two different types of artificial cranial deformation in two of the individuals. We combine bioarchaeological analysis with radiographic imaging, stable isotopes analysis, and ancient DNA to analyze their dietary patterns, molecular sex, and genetic affinities in the context of the archaeological data and their bioarchaeological attributes. While all three individuals were adolescent males with skeletal evidence of severe malnutrition and similar diets, the most striking observation is that they had major differences in their genetic ancestry. Results of the genetic analyses of the nuclear ancient DNA data for these individuals indicate that the individual without artificial cranial deformation shows broadly West Eurasian associated-ancestry, the individual with tabular oblique-type has East Asian ancestry and the third individual with circular erect-type has Near Eastern associated-ancestry. Based on these results, we speculate that artificial cranial deformation type may have been a visual indicator membership in a specific cultural group, and that these groups were interacting intimately on the Pannonian Plain during the Migration Period.

## Introduction

Humans exhibit similarities in cultural practices that span across space and throughout time. One such practice is Artificial Cranial Deformation (ACD), a widespread cultural phenomenon performed to denote group and/or individual identity; that is, to distinguish particular people from others or provide visible evidence of status, nobility, or affiliation to a certain class or group [1–4]. This practice of permanently shaping the cranial vault has been documented by anthropologists to have occurred on every inhabited continent, possibly dating back to the Late Paleolithic and practiced at least up to the 20^th^ century [5–10].

ACD is an irreversible and deliberate act performed by adults on infants, reflecting an investment of time and an effort to achieve an ascribed identity [1, 11]. Unlike other types of body modification, ACD is not associated with any other known rite of passage, making it an indelible symbol of social identity [11]. As such, it serves to foster intra-group solidarity and acts as a visual manifestation of inter-group cultural differences [11]. Taking advantage of the plasticity of the infant skull, ACD is carried out by application of constant external pressure on the head from the first days of life, often using boards, pads, bags of earth or clay, or specially-made headdresses, to achieve a desired shape [5, 12]. Because the cranium is a functional matrix comprising numerous interacting units, artificially-inflicted restriction of cranial growth along a particular axis will result in compensatory growth along a less restricted or unrestricted axis, ultimately causing an alteration of cranial shape [13–15]. Growth restriction of the cranial vault may also affect growth in adjacent areas, such as the cranial base or face [13, 14, 16–18]. While cranial morphology is affected, the brain enclosed inside the artificially deformed cranium achieves a volume comparable to that enclosed inside an unmodified cranium [15].

Historically, anthropologists have employed various classification systems for artificially-induced cranial deformations based on external vault morphology, though the definitions of deformation types are not consistent across the literature [14]. Some studies establish unique characteristic categories based on specific observations of the available samples, employing classification types such as “posterior flattening,” “bilobed,” and “circumferential” [19], or “occipital,” “coronal-occipital,” “occipital-parietal,” and “circular” [20]. Others emphasize more general morphological types, broadly comparing the anteroposteriorly-deformed type compared to the circumferentially-deformed type [5]. Both of these broad groups contain subgroups of “erecta” and “obliqua” types referring to the orientation of the deforming pressure, and therefore to the position of the occipital plane in relation to the facial plane. Specifically, the erect type deformation causes the occipital bone to take a direction perpendicular to the Frankfort plane, while the oblique type deformation causes the occipital to take a direction oblique in relation to the Frankfort plane [14, 21–23].

As important as classifying ACD by deformation type is understanding its origin and meaning in an archaeological context. The practice of ACD has ancient roots in Eurasia, appearing to arise independently at multiple points in time [1]. Archaeological evidence suggests that ACD on a broader scale was introduced to Central Europe with the migration of the Huns [2, 24]. During the Great Migration Period (5^th^/6^th^ century CE), this custom became widespread in the region of the Pannonian Plain (modern-day Austria, Croatia, Hungary, Romania, Serbia, Slovakia, and Slovenia), and was particularly popular among the Sarmatian, Alan, Gothic, Longobard, Gepidic, and Hun populations [4, 25]. The ACD detected in these populations has been categorized within the “Danube Basin Group” of Eurasian ACD, part of the six-group system based on archaeological, chronological, and geographic contexts by Nemeskéri [26]. Within this broad group, mainly represented by Hun and Germanic crania dated to the 5^th^/6^th^ centuries CE, the extent of deformation varies from slightly to heavily deformed and includes diverse types of modifications (i.e., different cranial shapes) [4]. Though detected across Pannonian Plain populations, the meaning of this cultural practice to these groups has not yet been clearly elucidated. While some earlier studies suggested that the deformed skull shape was initially a visible indication of nobility, and later became somewhat of a “trend” [27, 28], Molnár and colleagues more recently proposed that the custom of ACD in this region might be an indication of the social status [4].

Rescue archaeological excavations recently conducted at the well-known Hermanov vinograd site in Osijek, eastern Croatia, revealed a single pit, without any traces of the contemporary settlement, which contained a large number of commingled and fragmented human and animal skeletal remains together with the pottery fragments dated to the 5^th^ century CE, an era known as the Great Migration Period. Three partial human skeletons were discovered in this pit. Although the human remains belonging to the three individuals were haphazardly positioned without any consistencies in positioning and were only partially preserved, they all had complete crania. Analysis of the crania revealed that two of the individuals exhibited evidence of ACD, the first evidence of ACD detected in Croatia before the Avar Period. The discovery of this unusual archaeological feature and the unexpected characteristics of the remains found within it raised a number of questions about these individuals, primarily regarding their geographic origins and the potential meaning of ACD in this particular context.

Historic sources indicate that during the 5^th^ and 6^th^ centuries CE, the region around modern-day city of Osijek (the Roman province *Pannonia Secunda*) was under the Hunnish rule (until 454 CE), then under the Ostrogothic rule (till 473 CE), and finally under the Gepid rule, with some shorter interruptions, until 567 CE when they were replaced by the Avars [29]. The available archaeological data also support this hypothesis [30]. While archaeological information from Osijek does not clearly assign the individuals discussed in this study as belonging to one population or another, it is possible that these individuals were Huns, Ostrogoths or Gepids.

Recent studies of ACD have offered unprecedented insight into the origins of ACD, the biological and social correlates of this phenomenon, the long-term effects of this procedure on skeletal development and morphology, and details of ancient individuals who exhibit ACD, including their molecular sex and population genetic affinity. For example, a study by Cottin et al. aimed to reinvestigate the presumed effect of ACD on basicranial and masticatory elements by applying a 3D geometric morphometric approach to CT scans [31] while a study by Veeramah and colleagues [32] generated genomic data from 41 individuals with and without ACD that provided clear evidence of female-biased long-distance migration during the Migration Period in Europe. The mentioned studies led us to focus our investigation on the three individuals from Osijek by taking an approach which combines traditional bioarchaeological analysis with radiographic (CT) imaging, stable isotope analysis, and ancient DNA analysis to explore patterns associated with ACD at Osijek.

In this context, our present analysis of three archaeological individuals from the Hermanov vinograd site, two of whom exhibit ACD, has three aims: (i) determine the type of ACD in each individual; (ii) assess similarities and differences between the analyzed individuals in terms of age at death, sex, and overall health; and (iii) explore genetic affinities and components of ancestry in the studied individuals by means of ancient DNA analysis.

## Results

Though the skeletons were only partially preserved, the degree of preservation of the elements that were recovered during excavation was excellent. In the case of SU 259, an almost complete cranium with mandible, both scapulae, and most long bones (minus the left radius and ulna, and both fibulae) together with most of the ribs were present; SU 261 was represented by a complete cranium without the mandible, and coupled by the first ribs; and SU 750 was represented by a complete cranium with mandible, right scapula, five left and five right ribs, one thoracic and two lumbar vertebrae, the sacrum, the right innominate, and both femora. All three individuals were adolescents: estimated age at death for SU 259 was 14–16 years, for SU 261 was 12–16 years, and for SU 750 was 12–14 years. The skeletons exhibit several pathological changes: SU 259 had healed sub-periosteal new bone formation on both tibiae, SU 261 had healed porotic hyperostosis on both parietal bones, and SU 750 had healed porotic hyperostosis on both parietal bones and linear enamel hypoplasias on all anterior teeth. No traces of injuries (ante- or peri-mortem) or post-mortem cut-marks were recorded on any of the analyzed skeletons. The skeletal remains were relatively robust, and cranial morphology suggested that all three individuals were males. However, due to widely-known issues in sexing non-adult skeletons from both forensic and archaeological contexts based on metric and non-metric traits, we looked to confirm our sex assessment using results obtained by ancient DNA (aDNA) analysis.

**Fig 1:**
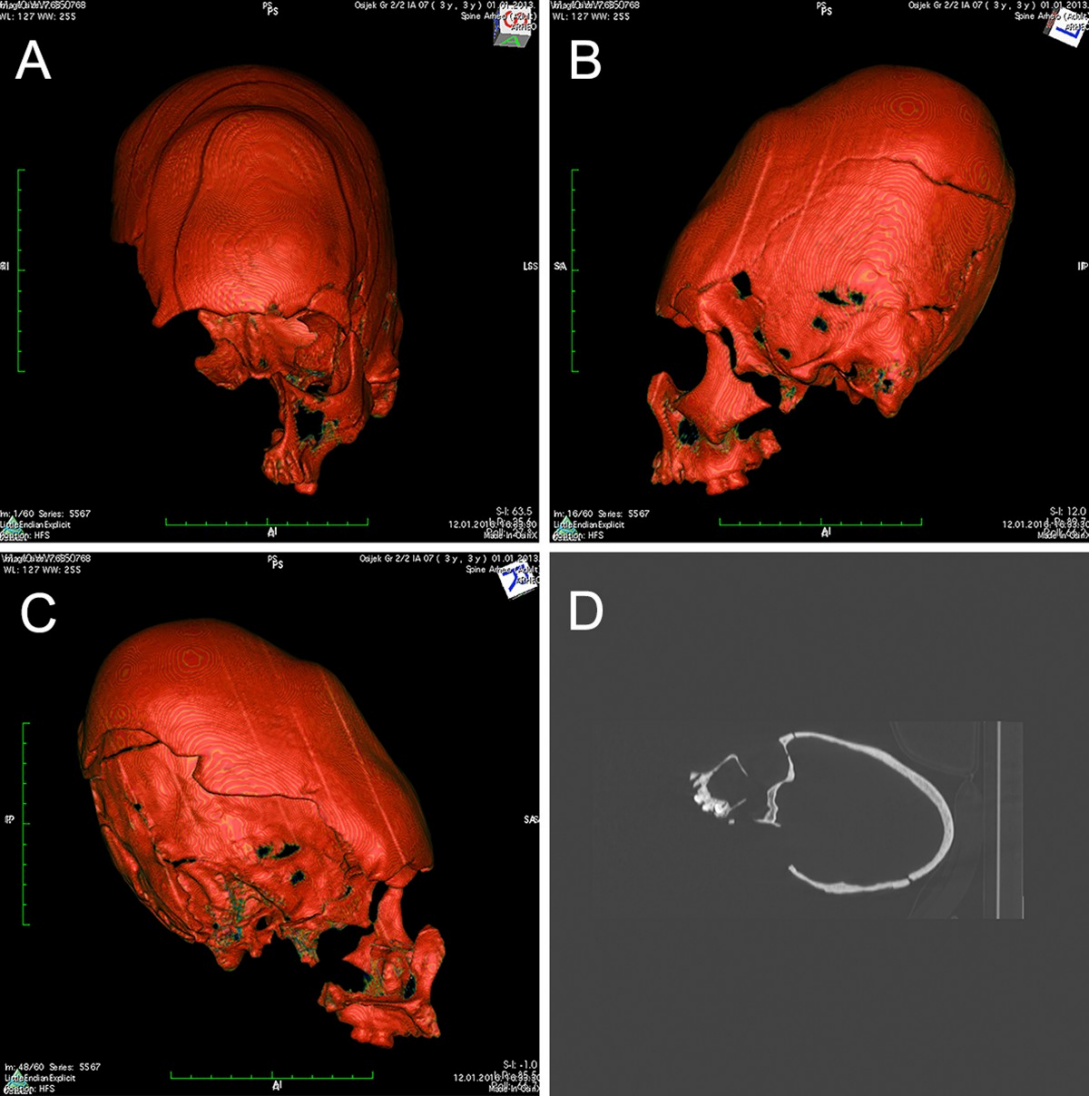
A-C) CT reconstruction showing the artificially deformed cranium belonging to individual SU 259, frontal and lateral views. The shape axis is dislocated posteriorly above the Frankfort horizontal plane while the cranium exhibits a depressed and strongly inclined frontal bone strongly indicating tabular oblique type of deformation. D) X-ray of the same cranium (lateral view) showing a significant thickening of the posterior part.

The cranium belonging to the individual SU 261 did not exhibit any traces of ACD, while morphological alternations of the crania consistent with ACD were recorded for SU 259 and SU 750. The cranial length of SU 259 is considerably increased and the general shape axis is dislocated posteriorly above the Frankfort horizontal plane (Fig 1A–1C). The cranium exhibits a depressed and strongly inclined frontal bone with a significant thickening of the posterior part visible on the anterior-posterior sagittal CT scan (Fig 1D); no traces of flattening are present on the occipital bone. The supraorbital ridges are almost non-existent, and all cranial sutures are completely fused as a result of the artificial modification. These observations suggest that SU 259 exhibits the tabular oblique type of ACD. It is widely accepted that the modifications resulting in tabular oblique deformation are carried out by the application of anterior-posterior compression using hard and rigid tools [3, 4]. In the case from Osijek, the modification was likely performed using a rigid tool in the frontal region only, as no traces of flattening are present on the occipital bone.

**Fig 2:**
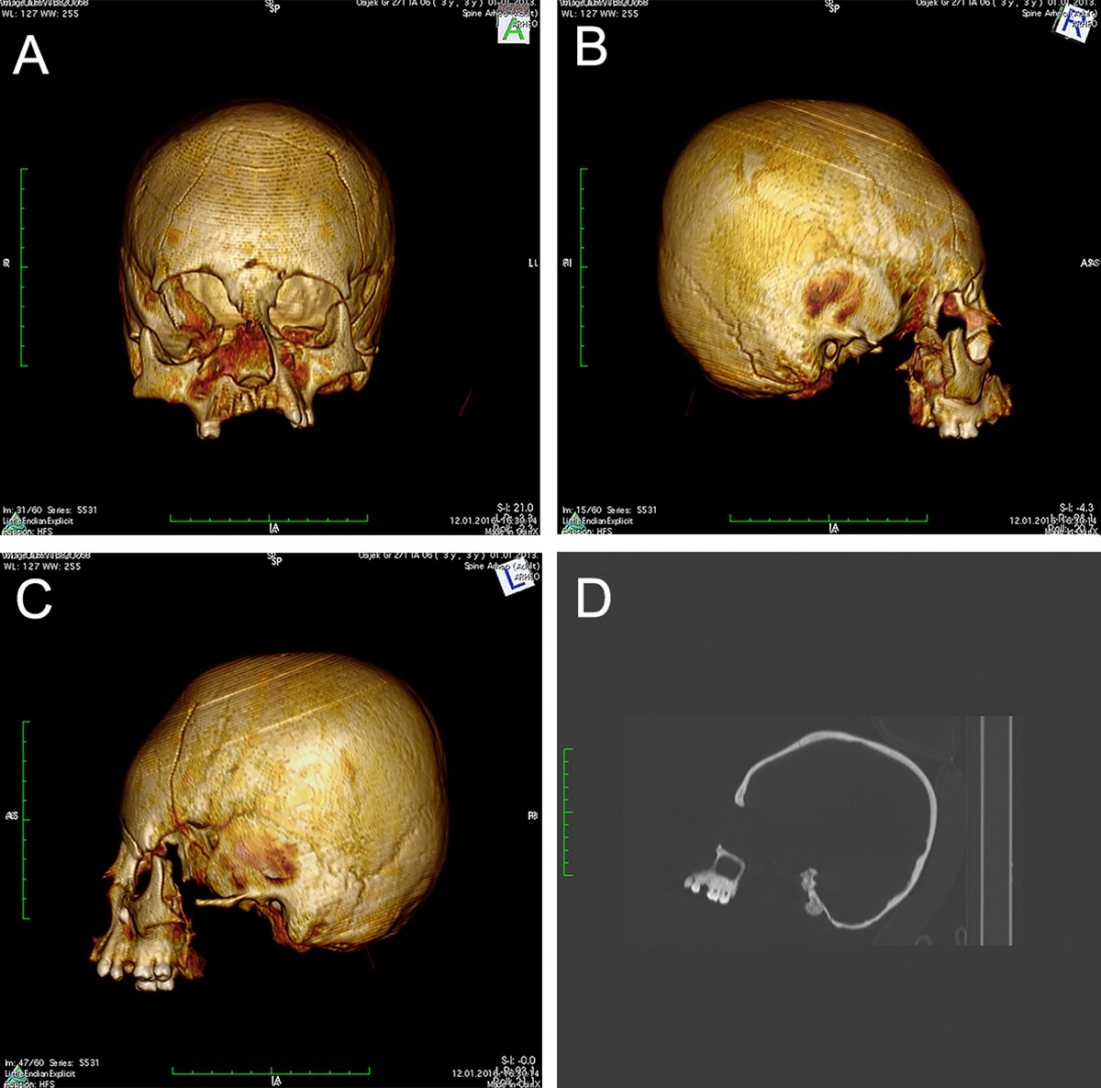
A-C) CT reconstruction showing the artificially deformed cranium belonging to individual SU 751, frontal and lateral views. A pronounced flattening of the frontal bone resulting in a remarkable growth of cranium height suggests the circular erect type of ACD. D) The flattening and significant thickening of the frontal bone anterior of bregma is visible on the X-ray image (lateral view).

In contrast, the cranium belonging to the individual SU 750 exhibits a pronounced flattening of the frontal bone resulting in a remarkable growth of cranium height. Approximately 30 mm anterior of bregma, the frontal bone is inclined at a 45-degree angle and is completely flattened up to about 60 mm superior of the supraorbital ridges (Fig 2A–2D). However, no similar changes were recorded on the occipital bone. These observations suggest that SU 750 exhibits the circular erect type of ACD. To achieve the circular erect deformation observed in SU 750, more flexible tools such as bandages, bands, tapes, and/or headdress were likely used [3, 4]. While it is possible that bandages were used to shape the cranium of SU 750, there are no macroscopically visible grooves caused by bandaging.

Analysis of carbon and nitrogen stable isotopes was performed on rib fragments belonging to all three individuals. While the bone sample from the individual SU 259 did not provide enough collagen to be analyzed, the samples from SU 750 and SU 261 did produce enough collagen and were analyzed as having C:N ratios of 3.2 (SU 750) and 3.3 (SU 261), indicating good protein preservation. Both had high δ^13^C values (SU 261 −17.0‰ and SU 750 −15.5‰) and very similar δ^15^N values (SU 261 9.5‰ and SU 750 9.9‰), suggesting a mixed terrestrial C3/C4 diet with a heavy reliance on resources such as millet, and a relatively low animal protein intake.

Authentic aDNA was recovered for all individuals (details presented in Table 1). Because only low-coverage shotgun sequencing was performed, approximately 500,000 human reads were identified for each individual, corresponding to endogenous DNA contents between 66–68%. The DNA sequences were examined for authenticity using deamination frequencies at the end of the DNA molecule and average sequence length. Deamination frequencies ranged between 15–17% on the 5’-end and 10–13% on the 3’-end of the DNA molecules, while sequence lengths averaged between 50–53bp with a standard deviation of 12–13bp (Table 1). Both of these metrics match the expected patterns for ancient DNA, which is predicted to exhibit a high frequency of DNA deamination at the terminal ends of the molecules and short sequence lengths due to chemical damage to DNA over time. Negligible human reads were detected in any of the blanks suggesting extremely low levels of cross-contamination. Between 4,086 and 5,838 SNPs were recovered for the three individuals (Table 1). The raw data has been deposited for public access in the European Nucleotide Database with the accession number PRJEB33257. Analysis of genetic sex was performed using a method that determines sex by considering the ratio of sequences aligning to the X and Y chromosomes [33]. Results were consistent with the assessment made using skeletal morphology, finding that all three individuals were males (Table 1).

**Table 1:** Sequencing results and sample details.

Sample	Total reads	Trimmed reads	Aligned reads	Aligned reads after duplicates removed	% endogenous	Average read length (standard deviation) (bp)	Genomic coverage	SNPs on the Human Origins dataset	Deamination frequencies of terminal bases (5' side | 3' side)	Molecular sex (X+Y to Y chromosome reads ratio)
ACD259	672857	536219	458219	457922	68.06	52 (12)	0.0077X	5838	0.15 | 0.11	Male (0.0896)
ACD261	494484	411303	326321	326122	65.95	50 (13)	0.0053X	4086	0.17 | 0.13	Male (0.0846)
ACD750	663984	512278	446365	446036	67.18	53 (12)	0.0077X	5774	0.15 | 0.10	Male (0.0906)
Extractions negative control	1031	803	9	9	0.87					
Library negative control	73	54	3	3	4.11					
PCR negative control	56	35	1	1	1.79					

**Fig 3:**
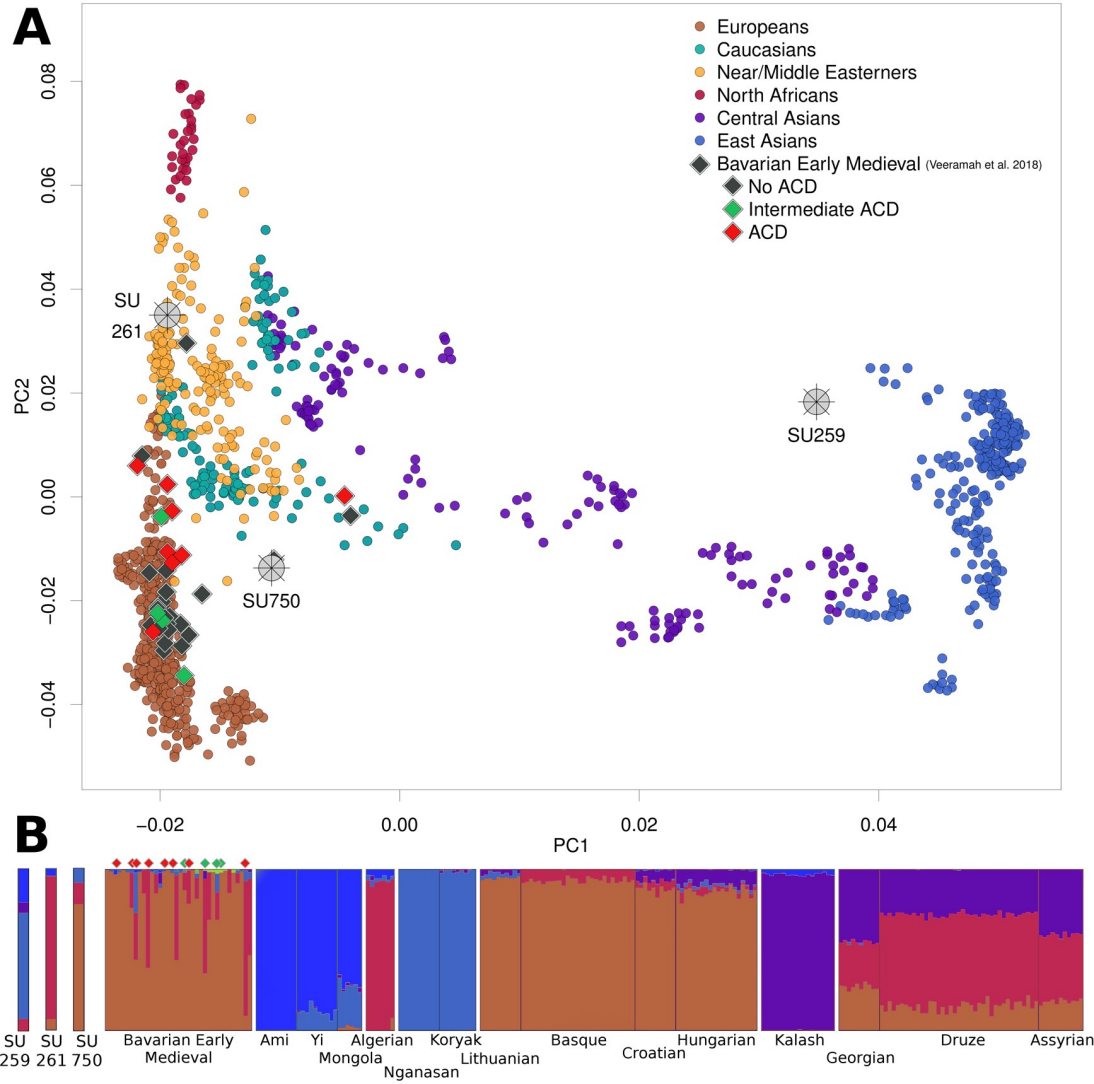
A) Principal component analysis of ancient samples (wheels and diamonds) projected onto variation of modern Eurasian and North-African populations. B) Model-based ancestry clustering with ADMIXTURE using K = 5 as the number of ancestral populations. Most informative modern populations where individual components were maximized are plotted.

The low number of SNPs used allowed us to associate individuals only with broad geographical areas when we performed PCA; however, PCA results clearly suggest starkly different population affinities among the individuals. SU 259 projects near to modern East Asian populations (the closest being Cambodian and Tu), while SU 261 overlaps with Near/Middle Eastern populations (represented, for example, by Palestinian, Syrian, and Lebanese), and SU 750 projects between populations from the Caucasus, Europe, and the Near/Middle East (Fig 3A). Though it lacks the power to elucidate detailed relationships between the individuals under study and with present-day populations, PCA provides a qualitative indication that, at this resolution, is able to distinguish populations in sub-continental groupings, demonstrating that these three individuals do not share close genetic affinities with each other.

With the intent of confirming that these results were not an artefact of low-resolution analysis, a secondary PCA was generated by reducing the pool of SNPs of the modern populations and the Early Medieval ancient individuals from Veeramah and colleagues [32] to the combined SNPs of our three individuals (totaling 15,698), and again projecting all ancient samples on to the PCs calculated using this dataset of reduced modern populations (S1 Fig). The distribution of the modern populations and the projected position of the three Osijek individuals were virtually unchanged, while the cluster of previously published Bavarian individuals expanded mostly on the PC1 axis, but while maintaining their West Eurasian affinities. This demonstrated that reducing the number of SNPs did not affect the sub-continental population groupings, and therefore suggests no significant analytical bias for these categorizations. Nevertheless, a conservative approach to this analysis should be taken, which is to consider both SU 261 and SU 750 as part of the West Eurasian cluster, and SU 259 part of the East Eurasian cluster.

**Fig 4:**
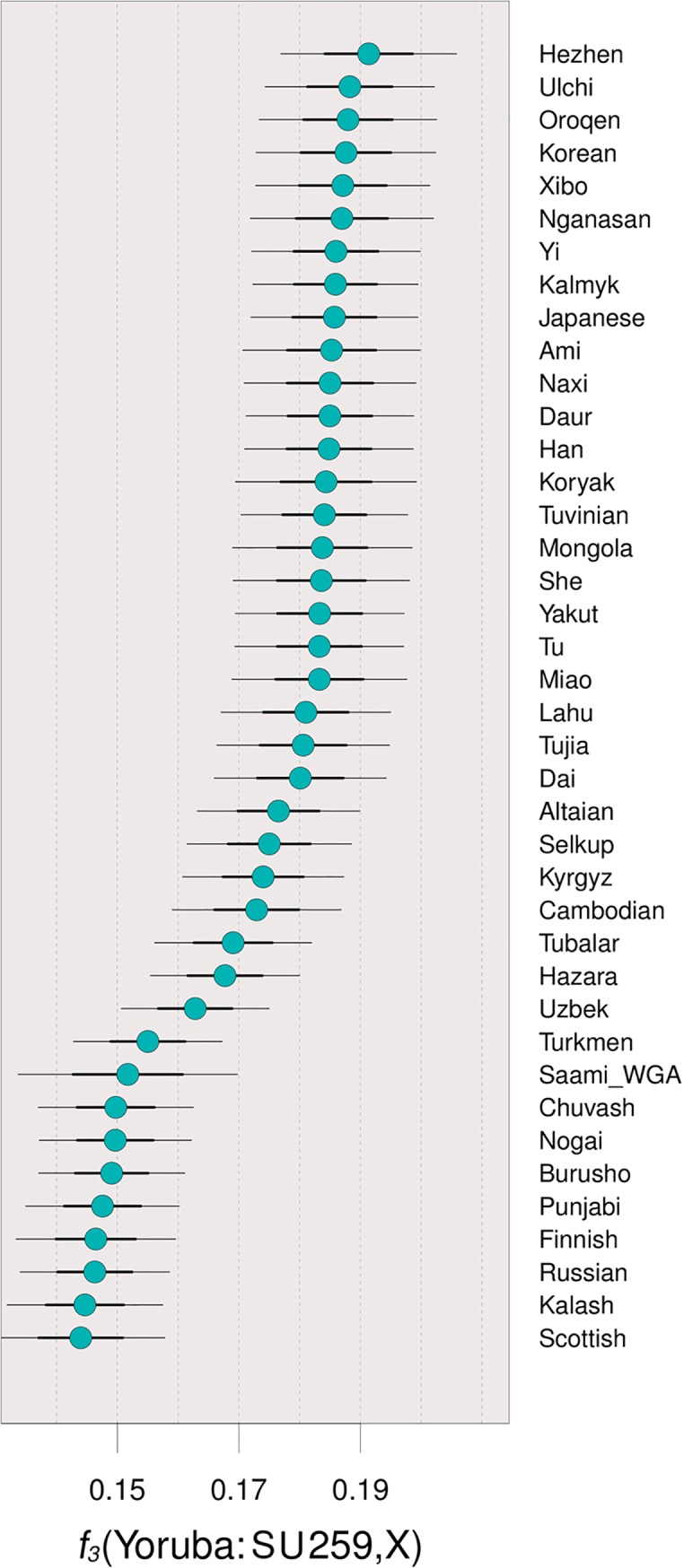
Outgroup-f3 results for individual SU 259 with thick and thin bars representing 1 and 2 standard deviations, respectively. The results show this individual shares higher affinities with East Asian than with West Eurasian, even when the standard deviations are considered.

The results from ADMIXTURE model-based clustering analysis are consistent with the differences in genetic affinities first detected using PCA (Fig 3A and 3B). Due to the low number of SNPs used in this analysis (ca. 5,000 SNPs per individual), only major ancestry components can be considered, as the small amounts of ancestry seen could be the result of statistical noise. Since cross-validation errors plateaued from K = 5 to K = 7, the replicate chosen was the one that maximized the main components seen on our three individuals, in this case at K = 5. ADMIXTURE results show that SU 259 primarily exhibits components of ancestry related to modern populations from East and Northeast Asia (including Ami, Mongola and Nganasan), consistent with the PCA results that suggested this individual shared the most affinity with populations from these regions (Fig 3B). The results of outgroup-*f*_*3*_ tests of the form *f*_*3*_(Yoruba, SU 259; East Asians/West Eurasians) confirm these affinities by showing that SU 259 shares the most alleles with East Asian populations such as Hezhen or Ulchi (Fig 4). Even when considering 2 standard deviations, the *f*_*3*_ estimates for West Eurasian populations do not overlap those for East Asia, providing further support for the Asian origin of SU 259. In contrast, individuals SU 261 and SU 750 do not exhibit significant amounts of East Asian ancestry. The main difference between these latter two individuals is the presence of a large component seen maximized in populations from Southeastern Europe, the Near/Middle East, and North Africa (including Algerian, Druze or Assyrian) in SU261, consistent with the PCA results that suggest this individual clusters closer to Near/Middle Eastern groups than to European groups (Fig 3A). In contrast, the most prominent component of SU 750 is one that is related to a component maximized in present-day continental European populations (including Lithuanian, Basque, or Hungarian). These latter two individuals show ancestries similar to the ones seen in the Early Medieval Bavarians from Veeramah and colleagues [32] (Fig 3B), which also included the presence of individuals of Southern European/Near Eastern ancestries.

## Discussion

Although the purpose of the pit excavated at Osijek—Hermanov vinograd remains unclear, we were able to successfully retrieve valuable biological information from the individuals whose remains were recovered from this pit.

As previously mentioned, all individuals shared notable similarities in terms of sex, age and general health: (i) all were males, (ii) all were adolescents between 12 and 16 years old at the time of death, and (iii) all displayed evidence of skeletal pathologies, including porotic hyperostosis, linear enamel hypoplasia and new sub-periosteal bone formation, though all of the lesions seemed to be inactive at the time of death.

The fact that all three studied individuals from Osijek were all adolescent males of almost the same age (between 12 and 16 years) may be suggestive of a cultural, regional, or temporal style of interment, though more data will be required to test this hypothesis. Though they appear to have been placed in the pit without clear and consistent positioning, it is possible that they were deposited with a certain intent rather than randomly disposed, as their remains were accompanied by faunal remains and pottery fragments. This style of interment and the characteristics of the remains recovered have also been seen in another Migration Period context; specifically, the sex and age of the Osijek individuals resemble the sex and age at death of the individual recovered from the Migration Period pit at the Ernei—Carieră site in Romania [34]. This feature in certain aspects resembles the case from Osijek (fragmented skeleton of a young male without any cut-marks in a pit accompanied by animal bones and pottery dated to the 4^th^/5^th^ c. CE); however, it also displays some differences such as the presence of numerous metal objects (weapons, tools and jewelry) and traces of burning [34]. Crișan and Lăzărescu characterized the feature from Ernei—Carieră as a ritual site “most probably involving beliefs regarding agricultural activities” [34]. Based upon the present state of research, it would be far-fetched to assume that the young males from Osijek and Ernei were human sacrifices; however, one has to bear in mind that certain cultures such as the ancient Moche of Peru preferred young adult males as their victims [35], and therefore, that this is a valid hypothesis that will benefit from future research.

The other striking similarity between the young males from Osijek is the fact that all three experienced ill-health during their childhood that was severe and lasting enough to be observed on their skeletal remains. Importantly, they all managed to survive and recover from physiological insults occurring during the early stages of their lives based on the healed state of these pathologies. Individuals SU 261 (no ACD) and SU 750 (ACD) display healed porotic hyperostosis on the cranial vault, a condition recognized by the occurrence of porosity on the outer table of the cranial vault (most often parietal bones and the occipital bone) and associated with acquired or genetic anemia caused by inadequate nutrition, metabolic or blood disorders, infectious disease, parasitism and weanling diarrhea [36–38]. SU 259 (ACD) displays healed sub-periosteal new bone formation on both tibiae, a woven bone formation that is macroscopically recognized as osseous plaques with demarcated margins or irregular elevations of bone surfaces [39]. The occurrence of this pathological change is usually associated with ‘non-specific infections’, but also with the conditions like birth trauma, metabolic disorders, hypervitaminosis A, leukemia, and infantile cortical hyperostosis [40]. Finally, SU 750 shows evidence of linear enamel hypoplasia which is an indicator of subadult stress usually recognized as one or more horizontal lines on the labial tooth surface. Due to the fact that these defects cannot be remodeled after the formation of enamel makes them excellent indicators of subadult stress in the first seven years of life [41, 42]. The most common explanations for the occurrence of linear enamel hypoplasia in the past are childhood disease and malnutrition [39, 43], poor sanitary conditions [44], and the effects of weaning [44, 45].

Based on the pathology data presented above, it is clear that the three individuals in question suffered from a number of long-term conditions, mostly associated with nutritional deficiency, during their childhood. According to the contemporaneous historic sources, such as Procopius and Enodius, frequent episodes of hunger and starvation were a common occurrence during the Migration Period in southern Pannonia [46]. Extended and severe periods of hunger were likely to have had a significant negative impact on the general health of the majority of population, as indicated by the results of conventional bioarchaeological studies conducted on skeletal remains recovered from several Late Roman/Migration Period sites in the region [47–49]. More specifically, these studies showed that at some sites, such as Zmajevac [47] and Štrbinci [48], approximately half of the analyzed skeletons exhibit evidence of linear enamel hypoplasia, cribra orbitalia/porotic hyperostosis and sub-periosteal new bone formation. It is therefore unlikely that these three individuals from Osijek were intentionally starved; instead, it is probable that other members of the community would have exhibited these same skeletal insults that suggest the embodiment of long-term stress.

Beside similarities in skeletal evidence of pathologies, similarities in diet were also identified; specifically, carbon and nitrogen stable isotopes analysis showed that at least two of the individuals (SU 261 and SU 750) had a mixed terrestrial C3/C4 diet with a heavy reliance on resources such as millet. This is almost identical to the results obtained by Hakenbeck and colleagues [50] in their study of dietary habits of the historically documented Huns and other nomadic groups from five Hungarian sites dated to the 5^th^ century CE. Similar results were obtained by the authors who studied diet of the somewhat later Avar populations from the region; for example, Noche-Dowdy [51], and Vidal Ronchas and colleagues [52] recorded almost identical values in several Avar sites from Hungary and Croatia dated to the 6^th^-9^th^ c. CE indicating a mixed diet containing C3 and C4 resources with a relatively low proportion of animal protein. Taken together, these studies suggest that C4 resources such as millet played the important role in everyday diet of various populations during the Migration Period in the region. This assumption is additionally strengthened by the presence of broomcorn millet in the nearby Bosnia and Herzegovina during this period [53], but also at the Avar site of Nuštar in eastern Croatia [54].

However, while they appear similar in terms of pathology and diet, two notable and potentially interrelated differences between these three individuals deserve further discussion and attempts at interpretation. First, two individuals from the same burial context at Osijek exhibit two different types of ACD. Second, distinct biogeographic ancestries are observed for the three individuals recovered from the pit.

The practice of ACD was particularly popular in Central Europe during the Migration Period, and so it is not unexpected that it would be identified at Osijek. However, while hundreds of cases of ACD from this region have been published, most of which have been associated with the Huns or various east Germanic tribes, including Gepids, Goths, and Alans [4, 25, 32, 55–57], only one case from Croatia, assigned to the Avar Period, has been published so far [58]. This makes our report of ACD in these individuals from Osijek very important in further elucidating the connection between Migration Period populations in Croatia and those in the rest of Central Europe. However, even more notable than the identification of ACD in the individuals from Osijek is that among the three individuals excavated from the same feature, two exhibited unique types of ACD. Specifically, SU 259 exhibited a type of ACD known as tabular oblique deformation, while the morphology of the cranium of SU 750 strongly suggested circular erect deformation [4].

As previously mentioned, both of these types of ACD are part of the “Danube Basin Group”, based on contexts by Nemeskéri [26]. The center of this group is estimated to be in present-day Hungary, but regional sub-centers corresponding to differing types and extent of deformation have been distinguished in the surrounding area [4]. In addition to Osijek, tabular oblique-type cranial deformation has been identified elsewhere on the Pannonian Plain during the Migration Period. Specifically, Molnár et al. [4] identify this type of ACD in the northeastern part of the Great Hungarian Plain in present-day Hungary at the site of Nyíregyháza-Rozsrétszőlő, a site possibly associated with Gepids or Huns. Circular deformation has been attributed to the Huns, who practiced this deformation type at a very high rate and spread its practice from the Ural Mountains to the Danube River following the Hun invasion of Eurasia [1]. Torres-Rouff and Yablonsky [1] report that Eurasian peoples, irrespective of their genetic origin and local customs, tried to emulate their prestigious Hun conquerors both culturally and physically, and that the circular-type deformation that arose as an element of Hun culture quickly lost its specific ethnic content, making it difficult to associate this type of deformation with a specific group. In addition to Osijek, erect cranial deformation has been identified at other sites on the Pannonian Plain during the Migration Period. For example, Molnár et al. [4] also identify this type of ACD at the site of Ároktő Csík-gát, where all individuals with ACD were of this type.

It remains unknown why two individuals who exhibit similarities in terms of sex and age at death, buried in the same context, would exhibit such different types of ACD. Upon identifying two distinct types of ACD in the Osijek individuals, we wondered if the distinct manifestations of ACD might have been a way of identifying the individuals as members of different cultural groups. We undertook ancient DNA analysis to further explore this idea, specifically using low-coverage shotgun sequencing data obtained using Next-Generation Sequencing to investigate the population affinities and biogeographic ancestry components of each individual. One of the individuals exhibiting ACD, SU 259, shows the greatest affinity to present-day Eastern Asians. This is the first case that we know of in which an individual from Migration Period Europe is shown to have major components of ancestry associated with present-day populations from East Asia, and expands the findings of Veeramah and colleagues [32], who identified possible components of East Asian ancestry in three Early Medieval Bavarian individuals, and Neparáczki et al. [59] who found mitogenomes of Asian origin in the conquering Hungarians of the 10^th^ century CE.

On the other hand, the individuals SU 261 and SU 750 exhibit components of ancestry close to modern Europeans and Near Easterners, respectively, a pattern of individual variation very similar to the individuals from Veeramah et al. [32]. The individual SU 261, who was estimated as having possible Southern European or Near Eastern ancestry is a male and did not show signs of ACD, clearly contrasting with the ACD female individuals found in Bavaria who had the same population affinity [32]. Further analysis of these individuals after deeper sequencing will enable higher precision population genomic analyses, such as admixture modelling, and will likely draw a clearer picture of these possible interactions.

## Conclusion

The presented study reports an unusual archaeological feature with human remains belonging to three individuals from eastern Croatia dated to the Migration Period. Based on the archaeological context and a direct radiocarbon date, the feature was dated to the 5^th^/6^th^ century CE and is likely associated with the Huns or Germanic tribes, a hypothesis supported by historical records. In addition to deriving from the same context, the three disarticulated and commingled skeletons found in the feature exhibit numerous similarities in biological terms: they belong to three adolescent males (between 12 and 16 years old at the time of death), they show similar pathologies suggesting a sustained and severe experience of stress, and finally, at least two individuals had a very similar diet. However, there is one significant morphological difference that distinguishes these individuals: two individuals show two different types of ACD (tabular oblique and circular erect deformations), while the cranium belonging to the third individual does not show any signs of artificial deformation. The results of genetic analyses point to an additional difference that cannot be discerned with the naked eye: these three individuals did not share the same biogeographic ancestry. More precisely, SU 259 (an individual with ACD) shows mainly East Asian ancestry, and is, to our knowledge, the first individual from the Migration Period with a majority of his ancestry originating in East Asia to be found in Europe. On the other hand, SU 750 (another individual with ACD) and SU 261 show West Eurasian ancestries similar to the patterns of population ancestry variation seen in Early Medieval Bavaria [32], though we note that our interpretations are made using low-resolution genomic data and are therefore conservative.

Although there are several hypotheses about the meaning of ACD in Pannonian Plain during the Migration Period, including an indication of social status or an indelible fashion statement, the cases from Osijek could suggest a different explanation. Based on the presented data, it seems that different types of ACD (or the lack thereof) might be associated with affiliation with a particular cultural group (at least in the case of Osijek), leading us to consider that populations with different ancestries and potentially different cultures were interacting intimately on the Pannonian Plain during the Great Migration Period. This leads us to ask: is this a random peculiarity or part of a larger-scale pattern of association between ACD type and group membership? We believe that future multi- and inter-disciplinary studies combining archaeology, bioarchaeology, history, stable isotopes analysis, and ancient DNA, conducted on a larger skeletal sample from a wider region will aid us in answering this question.

## Materials and methods

### Archaeological context

The human osteological remains presented in this paper were excavated in summer 2013 by Kaducej Ltd. at the Hermanov vinograd archaeological site in the city of Osijek in eastern Croatia (the exact geographic coordinates are 45° 32' 37'' N, 18° 40' 13'' E). While the 2013 excavations were a rescue campaign conducted as part of the construction of the Osijek southern bypass road [60], this famous archaeological site has been known since the end of the 19^th^ century when first archaeological excavations took place [61].

All necessary permits were obtained for the study, which complied with all relevant regulations. The permissions for the archaeological fieldwork and the scientific analyses of the human skeletal remains were issued by the Conservation Department of the Ministry of Culture of the Republic of Croatia in Osijek.

Hermanov vinograd is located in southwestern part of Osijek, and it is a predominantly Neolithic site associated with a Sopot culture settlement. The presence of several vertically-stratified construction phases suggest a period of active use that lasted for about 1,000 years [61]. More recent excavations at the Hermanov vinograd site revealed some archaeological features associated with later periods, mostly late Roman and early medieval times (5^th^/6^th^ century CE). One of these features was a circular pit (designated as burial 2), approximately 1.2 m in diameter and about 1.2 m deep. Pottery fragments from the feature suggested that it dated to the 5^th^ century CE (the Migration Period). No traces of a settlement associated with this pit were found, making it most likely that this pit was used by a group of nomadic people living in the area during this period.

**Fig 5:**
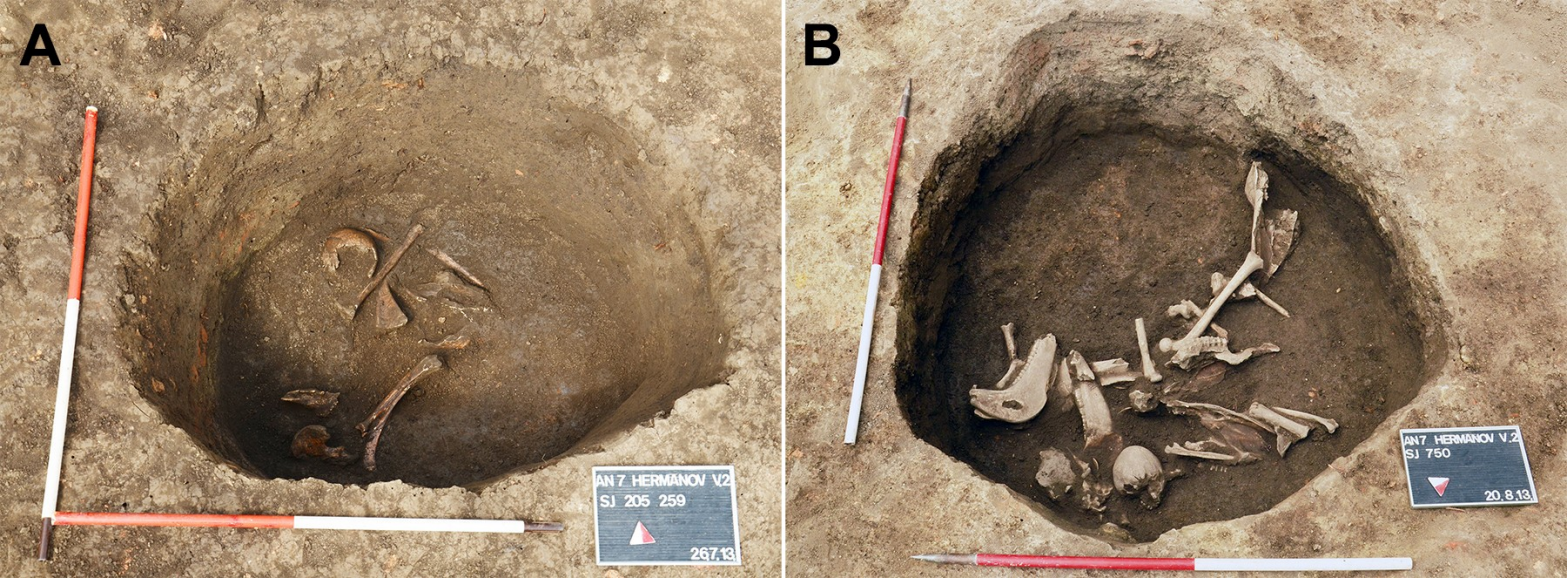
The pit from Osijek—Hermanov vinograd site during the excavation. A) The upper layer containing commingled skeletal remains, mostly human. B) The lower layer with commingled human and animal remains.

In addition to large quantities of animal bones and pottery fragments, mostly-disarticulated and partially-preserved human remains belonging to three individuals (SU 259, SU 261, and SU 750) were identified in the feature. Based on their lack of intentional positioning, the human remains appeared to be haphazardly deposited in the pit (Fig 5A and 5B). Radiocarbon dating of a human bone sample (Beta-435512) confirmed the use of this archaeological feature between 415 and 560 CE (Cal BP 1535 to 1390, 95% probability), consistent with the estimation made using pottery fragments, and confirming that the pit was in use during the Migration Period.

### Bioarchaeological analysis, radiographic imaging, and stable isotopes analysis

Conventional bioarchaeological analysis was conducted at the bioarchaeological laboratory of the Institute for Anthropological Research in Zagreb, Croatia. The skeletal remains are permanently stored at the Institute for Anthropological Research under reference number O-HV-1. Due to the disarticulated and partially-preserved nature of the remains, re-individualization of the skeletal remains was conducted prior to any other analysis. The re-individualization process was carried out using two types of data. The first is archaeological context, i.e. the position of the various skeletal elements in different layers of the pit. The second type of data used was taphonomic characteristics such as the state of preservation of the bone cortex as well as the coloring of the bone. Age at death was assessed for each individual using a frequently-utilized combination of skeletal features. This included the observation of changes occurring during the development and formation of deciduous and permanent teeth [62, 63], the degree of bone ossification [64], and the length of the diaphysis of long bones [65]. Also contributing to the estimation of age at death was an examination of the ectocranial suture fusion [66].

The biological sex of the three individuals was established based on macroscopic examination focusing on the differences in pelvic and cranial morphology between adult males and females [67–69]. All pathological changes were recorded according to criteria described by Aufderheide & Rodríguez-Martín [15], and Ortner [37]. For the purpose of this study, we used an ACD classification system previously proposed by Molnár et al. [4] in their analysis of Hun-Germanic (5^th^/6^th^ century CE) individuals from the Great Hungarian Plain. This classification system includes four different types of ACD: (i) tabular oblique, where cranial length and width both increase considerably and the general shape axis is dislocated posteriorly, (ii) tabular erect, where cranial height and width are increased as a result of anteroposterior compression and the general shape axis is approximately orthogonal in relation to the Frankfort horizontal plane, (iii) circular oblique, where a slight/pronounced flattening of the frontal and occipital bones and backward inclination in relation to the Frankfort plane is observed, and (iv) circular erect, where there is a slight or pronounced flattening of the frontal bone with a remarkable growth of the skull in height [4].

Radiographic imaging (CT scanning) of the two artificially deformed crania was conducted at the Department of Diagnostic and Interventional Radiology, University Hospital Dubrava in Zagreb, Croatia. The imaging was done utilizing a Multidetector computerized tomography (MDCT) unit (Emotion 16, Siemens AG Medical Solutions, Erlangen, Germany). Scanning parameters were 16 × 1.2 mm collimation and 1.0 reconstruction increment (RI) with 130 kV and 190 mAs respectively. Three-dimensional (3D) Volume Rendering Technique (VRT), Maximum Intensity Projection (MIP) and Multiplanar Reconstructions (MPR) were done with OsiriX MD Imaging software, v 7.0.4. (Pixmeo, Geneva, Switzerland).

Bone samples from the ribs of all three individuals were selected for the purpose of stable isotope analysis (nitrogen and carbon). Collagen for stable isotope analysis was extracted at University College Dublin, Ireland. Rib samples were demineralized in 0.5M HCl at 4C and then gelatinized in pH3 water at 70C for approximately 48 hours. The resulting solutions were filtered using Ezee filters prior to being freeze-dried. Carbon and nitrogen stable isotope analysis was conducted at University of Bradford Stable Light Isotope Laboratory, using a DeltaPLUS XL continuous flow isotope ratio mass spectrometer coupled via a ConFlo-III interface to a Thermo Flash EA 1112 elemental analyzer.

### Ancient DNA analysis

Bone powders from the cochlear part of the petrous of the three individuals were collected based on research showing that this the part of the skeleton preserves more endogenous DNA than other skeletal elements [70]. All bone processing, extraction, library preparation and quality control took place in dedicated ancient DNA facilities at University College Dublin, Ireland in adherence with strict anti-contamination protocols that included the physical separation of pre- and post-PCR spaces, a one-way rule for moving between ancient and modern laboratories, and the use of UV-irradiation in all working spaces [71–73].

DNA was extracted from the bone powder [74] and sequencing libraries were built for whole-genome shotgun sequencing [75]. Negative blanks were included at each step. The libraries from the three individuals were pooled into equimolar concentrations and sequenced together with the blanks on a single-end Illumina MiSeq (50-cycle v2 kit) run with a sequencing length of 65 base pairs (bp). The software cutadapt v1.1276 [76] was used to remove sequencing adapters allowing for a minimum overlap of 1 bp between the read and the adapter and minimum read length to 17bp, and bwa v.0.7.5a-r405 [77] was used to align the sequences to the human reference genome hg19, using a disabled seed and a minimum quality score of 30. The alignment files were then sorted and duplicate sequences were removed with the software samtools v0.1.19-96b5f2294a [78].

The genetic sex of each individual was assessed following the chromosome ratio method of Skoglund and colleagues [33], and single nucleotide polymorphisms (SNPs) were called using the Pileup tool of the Genome Analyzer Tool Kit’s (GATK) v.3.3-0-g37228af [79] based on the 616,938 autosomal genomic positions of the Human Origins dataset (retrieved from Lazaridis et al. [80]). For authenticity assessment, we calculated deamination frequencies of the terminal bases of the DNA sequences using the mapDamage2 software [81] as well as the average length of all sequences, recognizing that ancient DNA will be more damaged and fragmented into shorter molecules than modern DNA. The number of human reads in the sequenced negative blanks was also considered in our evaluation of authenticity and contamination. We tried to estimate contamination based on heterozygosity of the X-chromosome in male individuals using the software ANGSD but not enough sites were covered for such tests (median of 0).

For analysis of genetic affinity between our ancient individuals and other present-day and ancient populations of interest, we used the *smartpca* tool from the Eigensoft package [82], which projects ancient individuals onto a principal component analysis (PCA) calculated from modern Eurasian and North African populations. The modern populations consisted of the following, from [83, 84], and retrieved from [80]: Abkhasian, Adygei, Albanian, Algerian, Altaian, Ami, Armenian, Assyrian, Balkar, Balochi, Basque, Belarusian, Brahui, Bulgarian, Burusho, Cambodian, Canary_Islander, Chechen, Chuvash, Croatian, Cypriot, Czech, Dai, Daur, Druze, Egyptian, English, Estonian, Finnish, French, Georgian, Greek, Han, Hazara, Hezhen, Hungarian, Icelandic, Iranian, Iranian_Bandari, Italian_North, Italian_South, Japanese, Jordanian, Kalash, Kalmyk, Korean, Koryak, Kumyk, Kyrgyz, Lahu, Lebanese, Lezgin, Lithuanian, Makrani, Maltese, Miao, Mongola, Mordovian, Naxi, Nganasan, Nogai, North_Ossetian, Norwegian, Orcadian, Oroqen, Palestinian, Punjabi, Romanian, Russian, Saami_WGA, Sardinian, Saudi, Scottish, Selkup, She, Sicilian, Sindhi, Spanish, Spanish_North, Syrian, Tajik, Tu, Tubalar, Tujia, Tunisian, Turkish, Turkmen, Tuvinian, Ukrainian, Ulchi, Uzbek, Xibo, Yakut, Yi. As ancient individuals we used the contemporary ones from [32], who also exhibited cranial deformation. Although Amorim et al. [85] published ancient data from a similar period as the one studied in this manuscript we believe our current analysis benefits more from the data from Veeramah et al. [32] for the following reasons: a) no ACD has been described (or at least mentioned) in the two studied sites; b) the ancestry profiles of the individuals analyzed did not show any signs of East Asian ancestry (the one individual that possibly did, CL31, was found to have very high levels of contamination). We therefore believe Veeramah et al. [32] offers a more significant dataset considering the questions we tried to answer in our study. After pruning the dataset for linkage disequilibrium in the *plink* software with the option—*indep-pairwise 200 25 0*.*4*, unsupervised population structure was assessed with ADMIXTURE [86], for which we ran 5 replicates of the analysis for each K value (i.e., number of ancestral populations) between 2 and 8, and then examined the replicates with the lowest cross-validation error. Outgroup-*f*_*3*_ tests were used to estimate allele sharing between individuals/populations with the software *qp3Pop* from the AdmixTools package [83].

## Supporting information

S1 Fig Principal component analysis replicating Fig 4A on a reduced dataset composed of the combined 15,698 SNPs of the three studied individuals. (TIF)
