# Supplementary figures and images for: Cranial deformation and genetic diversity in three adolescent male individuals from the Great Migration Period from Osijek, eastern Croatia
Source: PLoS One. 2019 Aug 21;14(8):e0216366. doi: 10.1371/journal.pone.0216366 (PMC6703674; doi:10.1371/journal.pone.0216366)

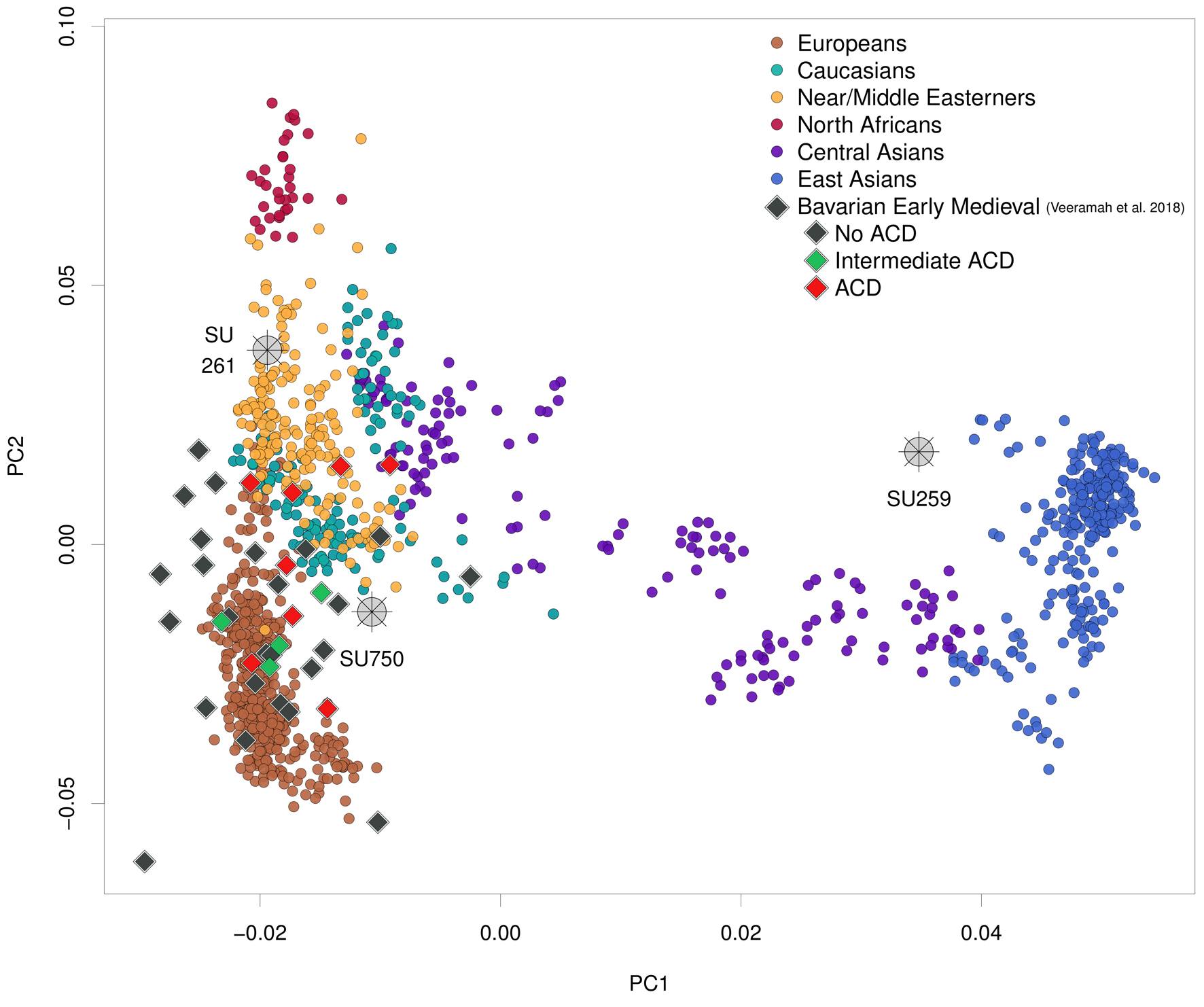

Supplement: S1 Fig — (TIF) [file pone.0216366.s001.tif]
